# Cigarette Smoke Extract-Induced Oxidative Stress and Fibrosis-Related Genes Expression in Orbital Fibroblasts from Patients with Graves' Ophthalmopathy

**DOI:** 10.1155/2016/4676289

**Published:** 2016-06-02

**Authors:** Hui-Chuan Kau, Shi-Bei Wu, Chieh-Chih Tsai, Catherine Jui-Ling Liu, Yau-Huei Wei

**Affiliations:** ^1^Department of Ophthalmology, Koo Foundation Sun Yat-Sen Cancer Center, Taipei 112, Taiwan; ^2^Department of Ophthalmology, Taipei Veterans General Hospital and National Yang-Ming University, Taipei 112, Taiwan; ^3^Department of Biochemistry and Molecular Biology, National Yang-Ming University, Taipei 112, Taiwan; ^4^Department of Medicine, Mackay Medical College, New Taipei City 252, Taiwan; ^5^Institute of Biomedical Sciences, Mackay Medical College, New Taipei City 252, Taiwan

## Abstract

Cigarette smoking is the most important risk factor for the development or deterioration of Graves' ophthalmopathy. Smoke-induced increased generation of reactive oxygen species may be involved. However, it remains to be clarified how orbital fibroblasts are affected by cigarette smoking. Our study demonstrated that Graves' orbital fibroblasts have exaggerated response to cigarette smoke extract challenge along with increased oxidative stress, fibrosis-related genes expression, especially connective tissue growth factor, and intracellular levels of transforming growth factor-*β*1 and interleukin-1*β*. The findings obtained in this study provide some clues for the impact of cigarette smoking on Graves' ophthalmopathy and offer a theoretical basis for the potential and rational use of antioxidants in treating Graves' ophthalmopathy.

## 1. Introduction

Graves' ophthalmopathy (GO), also called Graves' orbitopathy, thyroid-associated orbitopathy, or thyroid eye disease, is a cosmetically disfiguring and potentially vision-threatening disease. Although the pathophysiology of GO is still not fully clarified, it is known as a complex interplay process between multiple endogenous and environmental factors [1–4]. Cigarette smoking is the most important environmental and risk factor for the development or deterioration of GO, and the risk increases in parallel with the current number of cigarettes smoked per day [5–7]. Furthermore, cigarette smoking is associated with poor response to treatment for GO [8–10], and quitting smoking currently is the only method of GO prevention [11, 12]. In the study by Planck et al., some adipocyte-related immediate early genes, interleukin- (IL-) 1*β*, and IL-6 were overexpressed in smokers with severe active GO compared to nonsmokers, indicating that smoking activates pathways associated with adipogenesis and inflammation [13]. However, the exact mechanisms underlying the deleterious effect of smoking in GO remain to be identified. It has been proposed that smoke may induce the generation of reactive oxygen species (ROS) in the orbital socket, either through direct contact with the surrounding sinuses or indirectly through the blood circulation [14]. Cigarette smoke extract (CSE) has been reported to stimulate adipocyte differentiation in cultured orbital fibroblasts by synergizing with either IL-1 or ROS [12, 14]. In our previous study, we demonstrated that ROS could induce the protein expression of connective tissue growth factor (CTGF), an important fibrogenic factor, in cultured GO orbital fibroblasts [15]. The aim of the present study is to investigate the change of oxidative stress, fibrotic-related genes expression, and* intracellular cytokines* in the primary cultures of orbital fibroblasts in response to CSE. We also assessed whether or not CSE-induced oxidative stress, fibrotic-related genes expression, and* intracellular cytokines* in the GO orbital fibroblasts could be reduced by pretreatment of the cells with antioxidants.

## 2. Materials and Methods

### 2.1. Patients and Tissues Acquisition

The surgical specimens of 5 patients with GO (GO1–GO5) during orbital decompression surgery (one man and four women; mean age: 37 years) and the specimens of 5 age- and sex-matched patients (N1–N5) (one man and four women; mean age: 36 years) who received oculoplastic surgery for noninflammatory conditions were used in this study. All specimens were collected in accordance with the Declaration of Helsinki and with informed consent of the patients. All GO patients achieved stable euthyroidism with antithyroid medications for at least 6 months before surgery and are maintained in the inactive stage of GO. In addition, all study subjects had not received specific treatment (systemic steroids or radiotherapy) for GO. Exclusion criteria include ocular diseases other than GO, alcohol drinking, regular ingestion of antioxidants, and pregnancy. In addition, the patients suffering from chronic or acute diseases, such as diabetes mellitus, hyperlipidemia, diseases of the lung, liver, or kidney, cancer, other endocrine dysfunctions, and immunological or inflammatory disorders, were also excluded.

### 2.2. Primary Cultures of Orbital Fibroblasts

The primary cultures of orbital fibroblasts were established according to our previous study [16]. Briefly, the orbital tissues were minced aseptically in phosphate-buffered saline (PBS, pH 7.3) and then incubated with a sterile solution containing 0.5% collagenase and dispase (Sigma-Aldrich Chemical Co., St. Louis, MO, USA) for 24 hours in an incubator filled with an atmosphere of 5% CO_2_ and kept at 37°C. The mixture of digested orbital tissues was pelleted by centrifugation at 1,000 ×g and then resuspended in Dulbecco's Modified Eagle's Medium (DMEM, purchased from Gibco Life Technologies, Gaithersburg, MD, USA) containing 10% fetal bovine serum (FBS) and a cocktail of antibiotics (Biological Industries, Kibbutz Beit Haemek, Israel), which was composed of 100 U/mL penicillin G and 100 *μ*g/mL streptomycin sulfate (Biological Industries, Kibbutz Beit Haemek, Israel). The cultured orbital fibroblasts were used between the 3rd and 5th passages and the cell cultures at the same passage number were used for the same set of experiments.

### 2.3. Preparation of Cigarette Smoke Extract (CSE)

CSE was prepared according to prior studies with minor modification [12, 14]. The commercial cigarettes (The Longlife tobacco package purchased from Taipei, Taiwan) were used in this study, and each cigarette contained 10 mg tar and 0.8 mg nicotine. Ten pieces of cigarettes were smoked continuously by a pump-smoke machine, and this smoke was used to generate 200 mL of prewarmed CSE-PBS solution. Each cigarette was smoked for 3 min, and the pH of CSE-PBS solution was adjusted to 7.4 and then passed through a 0.22-*μ*m pore size filter (Millipore Corporation, Billerica, MA, USA) to remove large particulates and bacteria. The CSE-PBS solution is defined as 100% CSE, and this CSE will be diluted with DMEM in the following experiments. CSE preparation is standardized by measuring the absorbance at a wavelength of 320 nm (optical density = 2.0–2.2), and the pattern of absorbance observed at a wavelength of 320 nm shows insignificant variation between different preparations of CSE. CSE concentrations in the current study are ranged from 0 to 15%.

### 2.4. Treatment of Orbital Fibroblasts with CSE and Antioxidants

After washing with PBS buffer (pH 7.4) twice, the orbital fibroblasts were treated with various concentrations of CSE ranging from 1% to 15% for 24 hours. To investigate whether the effect of CSE could be blocked by antioxidants, we pretreated the orbital fibroblasts with 1 mM N-acetylcysteine (NAC) or 2 mM vitamin C (VitC) for 1 hour, followed by the induction of 5% CSE treatment for another 24 hours, respectively.

### 2.5. Determination of Cell Viability

Cell viability was measured by the Trypan blue exclusion assay and was counted by using a hemocytometer. The number of viable cells was determined on the basis of their exclusion of 0.4% Trypan blue (Sigma-Aldrich, St. Louis, MO, USA). The relative cell viability was normalized by the value of cells without CSE treatment and was expressed as mean ± SD of the results from three independent experiments.

### 2.6. Measurement of ROS Content

The probes from 2′,7′-dichlorofluorescin diacetate (DCFH-DA, Molecular Probes, Eugene, OR, USA) will be used to evaluate the intracellular H_2_O_2_ contents [17]. After incubation of orbital fibroblasts with 20 *μ*M DCFH-DA at 37°C for 20 min, cells were trypsinized and then resuspended in 0.5 mL of PBS buffer (pH 7.4) and analyzed with a flow cytometer (Model EPICS XL-MCL, Beckman-Coulter, Miami, FL, USA). The excitation wavelength is set at 488 nm and the intensity of emitted fluorescence of a total of 10,000 cells at 525 nm is recorded on channel FL1 for the DCFH-DA probe. Data were analyzed by the EXPO32*™* software (Beckman-Coulter, Miami, FL, USA). The intracellular H_2_O_2_ contents in the treated cells were presented as relative values compared with that of the cells without H_2_O_2_ or CSE treatment.

### 2.7. Determination of Lipid Peroxidation

The lipid peroxidation product, malondialdehyde (MDA), in cultured orbital fibroblasts was measured by the spectrophotometric assay kit (MDA-586; OxisResearch Inc., Portland, OR, USA) according to the manufacturer's instructions [18]. The MDA is quantified in the reaction with a chromogenic regent N-methyl-2-phenylindole to form an intensely colored carbocyanine dye with a maximum absorbance at 586 nm. The method is specific for MDA instead of other lipid peroxidation products such as 4-hydroxyalkenal because they cannot produce significant absorbance at 586 nm under the experimental conditions. An MDA standard curve was established by using the MDA samples at the concentration range of 0–50 *μ*M, and the MDA levels in orbital fibroblasts were normalized to cell numbers (10^6^ cells). The results were expressed as mean ± SD of the results from three independent experiments.

### 2.8. Western Blot Analysis

An aliquot of 50 *μ*g proteins was separated on 10% SDS-PAGE and blotted onto a piece of the PVDF membrane (Amersham-Pharmacia Biotech Inc., Buckinghamshire, UK). After blocking by 5% skim milk in the TBST buffer (50 mM Tris-HCl, 150 mM NaCl, and 0.1% Tween 20, pH 7.4) at room temperature for 1 hour, the membrane was incubated for another 1 hour with the primary antibody at room temperature. After washing three times with the TBST buffer, the membrane was incubated with a horseradish peroxidase- (HRP-) conjugated secondary antibody for another 1 hour at room temperature. An enhanced chemiluminescence detection kit (Amersham-Pharmacia Biotech Inc., Buckinghamshire, UK) was used to detect the protein signals with a Fuji X-ray film (Fuji Film Corp., Tokyo, Japan), and the intensities of signals were quantified by ImageScanner III with LabScan 6.0 software (GE Healthcare BioSciences Corp., Piscataway, NJ, USA). The antibodies of HO-1 (SC-10789) and CTGF (SC-14939) were purchased from Santa Cruse Biotechnology Inc. (CA, USA), and *β*-actin (#A1978) was purchased from Sigma-Aldrich Chemical Co. (MO, USA), respectively. All data were expressed as mean ± SD of the results obtained from three independent experiments.

### 2.9. Real-Time Reverse Transcriptase-Polymerase Chain Reaction (RT-PCR)

The expression levels of fibrosis-related genes were determined by SYBR green-based real-time quantitative PCR. Briefly, the total cellular RNA from orbital fibroblasts lysates was extracted with a chloroform solution after adding the TRIZol reagent (MO, USA). The extracted RNA was precipitated with isopropanol solution, dried on ice, and dissolved in DEPC-H_2_O. An aliquot of 5 *μ*g RNA was reverse-transcribed to cDNA with the Ready-to-Go RT-PCR kit (Amersham Biosciences, Uppsala, Sweden) at 42°C overnight. Quantitative RT-PCR was performed using the SYBR Green Master kit (Sigma-Aldrich) according to the manufacturer's instructions [18]. The primer pairs were 5′-CTCAACACGGGAAACCTCAC-3′ and 5′-CGCTCCACCAACTAAGAACG-3′ for 18S rRNA, 5′-CTGCAGGCTAGAGAAGCAGAG-3′ and 5′-GATGCACTTTTTGCCCTTCT-3′ for CTGF, 5′-CTGGCCGAAAATACATTGTAA-3′ and 5′-CCACAGTCGGGTCAGGAG-3′ for fibronectin, and 5′-GGACATCCACTTCCACAGC-3′ and 5′-GGTCATCGTCGCCTTCTC-3′ for apolipoprotein J, respectively. The mRNA expression level of each gene in the orbital fibroblasts was normalized with the mRNA level of the 18S rRNA gene, respectively.

### 2.10. Measurement of the Intracellular Cytokine Content

The human transforming growth factor-beta 1 (TGF-*β*1; catalog #DB100B) and interleukin-1*β* (IL-1*β*; catalog #DLB50) levels in cell culture supernatant were quantified with enzyme-linked immunosorbent assay kits purchased from R&D Systems, Inc. (Minneapolis, MN). Briefly, about 10^5^ orbital fibroblasts were seeded in a 3.5-cm culture dish and incubated for 48 hours at 37°C in a cell incubator with an atmosphere of 5% CO_2_ followed by treatment of 5% CSE for another 24 hours, or the cells were pretreated with 1 mM NAC or 2 mM VitC for 1 hour followed by the induction of CSE treatment for another 24 hours, respectively. According to the manufacturer's recommendation and our previous study [19], cell culture supernatant was centrifuged at 12,000 ×g at 4°C, and the aliquots were immediately assayed. The standards for TGF-*β*1 and IL-1*β* were used in a range of 0–200 pg/mL, and the results were normalized by the cell number and expressed as pg/10^4^ cells.

### 2.11. Statistical Analysis

The Microsoft Excel 2010 statistical package and SigmaPlot software version 12.3 (Systat Software Inc., San Jose, CA, USA) were used to analyze the results, and data were presented as means ± standard deviation (SD) of the results obtained from three independent experiments. The significance level of the difference between the control and the experimental groups was determined by Student's *t*-test. A difference was considered statistically significant when the ^*∗*^ *p* value < 0.05 and ^*∗∗*^ *p* value < 0.01, respectively.

## 3. Results

### 3.1. CSE-Induced Cytotoxicity and Oxidative Stress in the Orbital Fibroblasts

**Figure 1:**
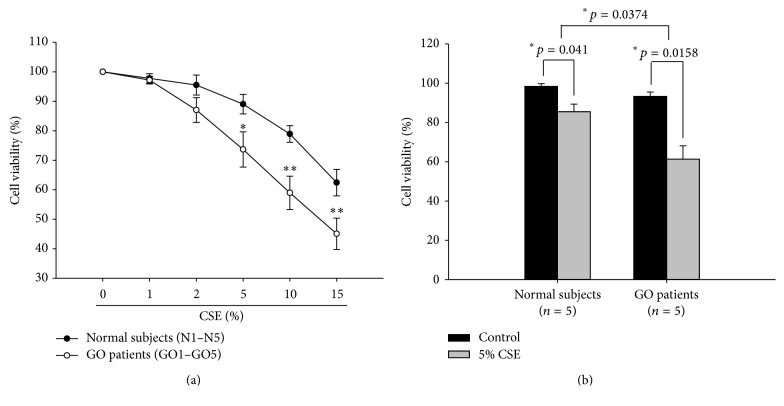
Susceptible to cigarette smoke extract- (CSE-) caused cytotoxicity in the GO orbital fibroblasts as compared to those of normal controls. (a) After treatment of orbital fibroblasts from GO patients (GO1–GO5) and age-matched normal subjects (N1–N5) with various concentrations of CSE ranging from 0 to 15% for 24 hours, the cell viability was determined by the Trypan blue exclusion assay. (b) The mean values of cell viability by 5% CSE treatment were shown in the histogram, and data were presented as means ± SD of the results from three independent experiments (^*∗*^ *p* < 0.05; ^*∗∗*^ *p* < 0.01 versus the indicated group).

**Figure 2:**
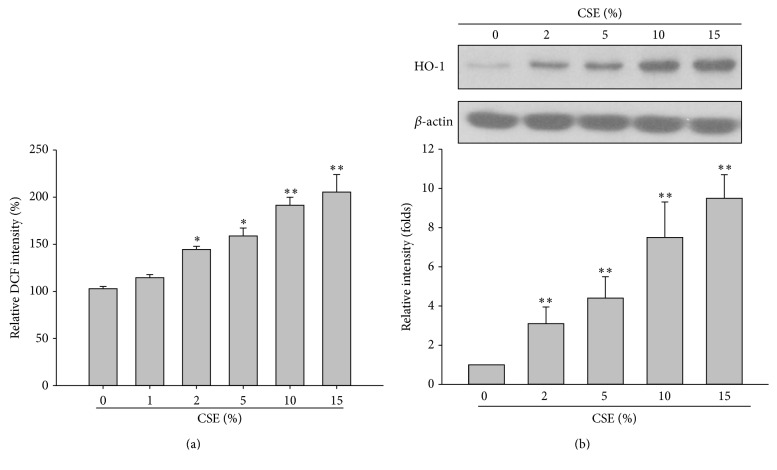
CSE-induced oxidative stress and oxidative response in a dose-dependent manner in the GO orbital fibroblasts. (a) After treatment of GO orbital fibroblasts with various concentrations of CSE ranging from 0 to 15% for 24 hours, the intracellular ROS was measured by DCF staining with a flow cytometer, and (b) the HO-1 protein expression was determined by Western blot. The representative histogram was constructed on the basis of the results from three independent experiments, and data were presented as means ± SD (^*∗*^ *p* < 0.05; ^*∗∗*^ *p* < 0.01 versus the control group without CSE treatment).

In order to investigate the cytotoxic effect of smoke extracts in the orbital fibroblasts, we treated the orbital fibroblasts with various concentrations of CSE for 24 hours. The effect of CSE ranging from 0 to 15% on the viability of the orbital fibroblasts from patients with GO (GO1–GO5) and normal subjects (N1–N5), as determined with the Trypan blue exclusion assay, is illustrated in Figure 1. The data show that both normal and GO fibroblasts were reduced in a dose-dependent manner, respectively (Figure 1(a)). The difference in cell viability between normal and GO orbital fibroblasts was statistically significant upon treatment with 5% CSE (Figure 1(b), 85% versus 62%, *p* = 0.0374). On the other hand, we also observed the CSE-induced oxidative stress and oxidative response in the GO orbital fibroblasts. After treatment of GO orbital fibroblasts with various concentrations of CSE (0–15%) for 24 hours, the intracellular ROS measured by DCF staining with a flow cytometer and the heme oxygenase-1 (HO-1) protein expression with Western blot were both increased in a dose-dependent manner (Figure 2).

### 3.2. Susceptible to 5% CSE-Induced Oxidative Stress and Oxidative Damage in the GO Orbital Fibroblasts as Compared to Those of Normal Controls and the Protective Role of Antioxidants

**Figure 3:**
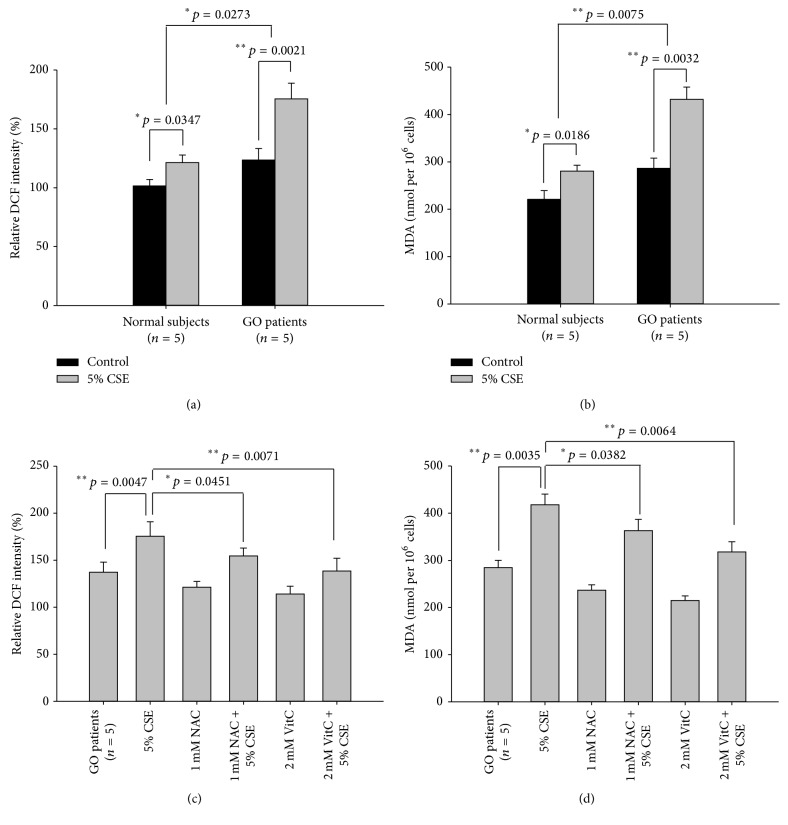
Susceptible to CSE-induced oxidative stress and oxidative damage in the GO orbital fibroblasts as compared to those of normal controls and the role of antioxidants. (a) After treatment of orbital fibroblasts from GO patients (GO1–GO5) and normal subjects (N1–N5) with 5% CSE for 24 hours, the intracellular ROS and (b) MDA levels were determined as described in Materials and Methods. (c) After pretreatment of GO orbital fibroblasts with 1 mM NAC or 2 mM vitamin C (VitC) for 1 hour followed by the addition of 5% CSE for another 24 hours, the intracellular ROS and (d) MDA levels were determined. The results were from three independent experiments, and data were presented as means ± SD (^*∗*^ *p* < 0.05; ^*∗∗*^ *p* < 0.01 versus the indicated group).

Due to the fact that the exposure of 5% CSE could significantly reduce the cell viability in the GO orbital fibroblasts as compared to those in the normal controls, we decided to treat normal and GO orbital fibroblasts with 5% CSE in the following experiments. After treatment of orbital fibroblasts with 5% CSE for 24 hours, the intracellular ROS measured by DCF staining was significantly increased in both normal and GO orbital fibroblasts, respectively (Figure 3(a), *p* = 0.0347 and *p* = 0.0021). In addition, the lipid peroxidation marker, malondialdehyde (MDA), was also significantly increased in both normal and GO orbital fibroblasts, respectively, after the addition of 5% CSE for 24 hours (Figure 3(b), *p* = 0.0186 and *p* = 0.0032). Moreover, we noted that the induction ratio of intracellular ROS and MDA levels after treatment with 5% CSE was more pronounced in the GO orbital fibroblasts than those in the normal controls, respectively (Figures 3(a) and 3(b), *p* = 0.0273 and *p* = 0.0075). On the other hand, we also observed the protective effects of NAC and VitC on CSE-induced oxidative stress and oxidative damage in the GO orbital fibroblasts, respectively. Preincubation with 1 mM NAC or 2 mM VitC for 1 hour, respectively, significantly decreased 5% CSE-induced elevations of intracellular ROS measured by DCF staining in the GO orbital fibroblasts (Figure 3(c), *p* = 0.0451 and *p* = 0.0071). A significant reduction in 5% CSE-induced elevations of MDA contents was also obtained after the GO orbital fibroblasts were preincubated with 1 mM NAC or 2 mM VitC, respectively (Figure 3(d), *p* = 0.0382 and *p* = 0.0064).

### 3.3. Susceptible to 5% CSE-Induced Changes of Fibrosis-Related Genes Expression in the GO Orbital Fibroblasts as Compared to Those of Normal Controls

Previously, we have shown that the elevated intracellular oxidative stress was associated with the increase of fibrosis-related genes expression in the GO orbital fibroblasts [15]. In this study, we further investigated whether 5% CSE-induced ROS could lead to inducing the fibrosis-related genes expression in the orbital fibroblasts. By a SYBR green-based RT-PCR, we observed the significant elevation in the levels of fibrosis-related genes expression including apolipoprotein J, fibronectin, and CTGF in both normal and GO orbital fibroblasts after treatment of 5% CSE for 24 hours (Table 1, *p* = 0.0431 versus *p* = 0.0085, *p* = 0.0318 versus *p* = 0.0033, and *p* = 0.0441 versus *p* = 0064, resp.). In addition, the induction ratio of apolipoprotein J, fibronectin, and CTGF by 5% CSE was more pronounced in the GO orbital fibroblasts than those in the normal controls (Table 1, *p* = 0.0086, *p* = 0.0031, and *p* = 0.0054, resp.).

### 3.4. Inhibition of 5% CSE-Induced Fibrosis-Related Genes Expression by Antioxidants in GO Orbital Fibroblasts

**Figure 4:**
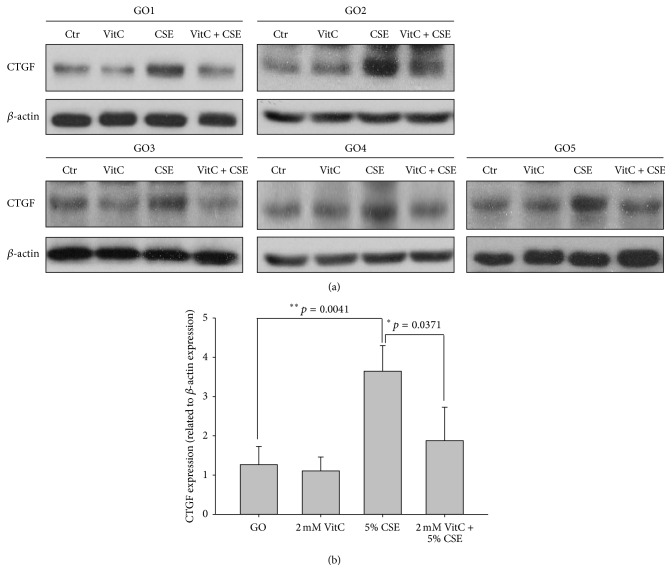
Inhibition of CSE-induced fibrotic markers by pretreatment of the GO orbital fibroblasts with antioxidants. (a) After pretreatment of orbital fibroblasts from GO patients (GO1–GO5) with 2 mM VitC for 1 hour followed by the addition of 5% CSE for another 24 hours, the CTGF protein expression was determined by Western blot, respectively. (b) The levels of the CTGF protein expression were normalized to each of corresponding *β*-actin expression levels and were adjusted to GO1 without CSE and/or vitamin C (VitC) treatment, whose CTGF expression was defined as 1.00. The representative histogram was constructed on the basis of the results from three independent experiments, and data were presented as means ± SD (^*∗*^ *p* < 0.05; ^*∗∗*^ *p* < 0.01 versus the group without CSE treatment). Ctr: without CSE treatment.

To investigate whether 5% CSE-induced fibrosis-related genes expression in GO orbital fibroblasts could be blocked by antioxidants, we pretreated the orbital fibroblasts with 1 mM NAC or 2 mM vitamin C, respectively, for 1 hour followed by the 5% CSE treatment. The results showed that preincubation of cells with 1 mM NAC or 2 mM VitC could significantly inhibit the 5% CSE-induced fibrosis-related genes expression including apolipoprotein J, fibronectin, and CTGF in the GO orbital fibroblasts by a SYBR green-based RT-PCR (Table 2). The inhibition ratio by 1 mM NAC treatment for apolipoprotein J, fibronectin, and CTGF is 14%, 17%, and 13%, respectively (*p* = 0.0437, *p* = 0.0251, and *p* = 0.0470, resp.). The inhibition ratio in the GO orbital fibroblasts by 2 mM vitamin C treatment for apolipoprotein J, fibronectin, and CTGF is 24%, 28%, and 27%, respectively (*p* = 0.0294, *p* = 0.0085, and *p* = 0.0224, resp.). Accordingly, we also examined CSE-induced expression levels of CTGF protein by Western blot. The result showed that the protein expression of CTGF was significantly increased in the GO orbital fibroblasts after the addition of 5% CSE for 24 hours (Figure 4, *p* = 0.0041). Besides, the pretreatment of GO fibroblasts with 2 mM VitC could also inhibit 43% of the elevations in the CTGF protein expression (Figure 4, *p* = 0.0371).

### 3.5. Susceptible to 5% CSE-Induced Changes of Intracellular Cytokines in the GO Orbital Fibroblasts as Compared to Those of Normal Controls and the Protective Role of Antioxidants

The changes in the intracellular cytokines after stimulation of orbital fibroblasts with 5% CSE are shown in Table 3. Basal levels of TGF-*β*1 and IL-1*β* were significantly higher in the GO orbital fibroblasts as compared to those of the normal controls (*p* = 0.004 and *p* = 0.008, resp.). In addition, CSE induced significant increase in TGF-*β*1 and IL-1*β* levels in GO orbital fibroblasts as compared to the respective controls (*p* = 0.006 and *p* = 0.005, resp.). Moreover, the induction ratio of TGF-*β*1 and IL-1*β* after stimulation with 5% CSE was more pronounced in GO orbital fibroblasts than those in the normal controls (*p* = 0.008 and *p* = 0.003, resp.).  Table 4 showed a significant reduction in 5% CSE-induced elevations of intracellular TGF-*β*1 and IL-1*β* after the GO orbital fibroblasts were pretreated with 1 mM NAC (*p* = 0.037 and *p* = 0.028, resp.) or 2 mM VitC (*p* = 0.008 and *p* = 0.003, resp.).

## 4. Discussion

Evidence is mounting that oxidative stress plays an important role in the development of GO [17, 20–22]. We demonstrated in this study that CSE elicited more pronounced response of oxidative stress in GO orbital fibroblasts. More importantly, this is the first study to reveal that CSE could induce fibrosis-related genes expression, especially CTGF, in the GO orbital fibroblasts as compared with those of normal controls. In addition, pretreatment with antioxidants such as NAC and vitamin C could confer significant protection against the influence of CSE on oxidative damage, fibrosis-related genes expression, and induction of TGF-*β*1 and IL-1*β*.

Orbital fibroblasts, one of the major target cells in GO, are associated with many GO-related pathologic conditions, including oxidative stress [14, 15, 23]. Oxidative stress also has been suggested to play a role on the deleterious impact of smoking in GO [14]. The present study provided evidence that the GO orbital fibroblasts were more susceptible to CSE-induced cytotoxicity and oxidative stress than those of normal controls. Accumulation of CSE-induced ROS may cause more oxidative damage including oxidative DNA damage and lipid peroxidation, which could explain in part our previous observation that smokers had significant higher urinary 8-hydroxy-2′-deoxyguanosine (8-OHdG) than did never smokers in GO patients [24]. In addition, increased generation of ROS, especially the superoxide anions and hydrogen peroxide, can stimulate proliferation of GO orbital fibroblasts [19] and induce the production of proinflammatory cytokines [25], which all are key pathological features in GO. Moreover, cigarette smoke-mediated oxidative stress could recruit inflammatory and immune cells such as lymphocytes, macrophages, and neutrophils and activate some proinflammatory mediators [26], which may exacerbate the inflammation and tissue remodeling processes of GO.

The disease course of GO is characterized not only by early inflammatory process but also by tissue remodeling and/or fibrosis. Although fibrosis represents a quiescent stage in GO, it may cause much of the substantial morbidity, which is often unresponsive to conventional medical treatment and requires surgical intervention. Oxidative stress is known as a factor that can induce various pathological fibrosis [27, 28]. In current study, we also noted that cigarette smoke-mediated oxidative stress could induce fibrotic-related genes expression including apolipoprotein J, fibronectin, and CTGF in the GO orbital fibroblasts, and these effects could be inhibited by pretreatment with antioxidants. Apolipoprotein J, fibronectin, and CTGF are commonly known as important fibrogenic factors. Although it remains to be clarified whether apolipoprotein J plays as a fibrosis biomarker or adaptive response in the development of fibrotic process of GO, CTGF has been shown to be substantially involved in the pathogenesis of various fibrotic disorders such as liver, heart, kidney, and ocular fibrosis [29–32]. CTGF can exhibit diverse cellular functions, including extracellular matrix production, cell migration, proliferation, and differentiation. Importantly, CTGF is critical for TGF-*β*-mediated fibroblast-myofibroblast transdifferentiation and subsequent deposition of extracellular matrix [33], which may contribute to the tissue remodeling and fibrosis process in GO. It has also been reported that periodontal fibrosis can be promoted by nicotine from smoking via effects on CTGF [34]. Taken together, previous reports and our findings in this study may explain in part why smoking is associated with severe GO and poor response to immunosuppressive therapy in GO.

We previously revealed that low concentrations of hydrogen peroxide can induce the production of proinflammatory cytokines such as TGF-*β*1 and IL-1*β* in GO orbital fibroblasts [19]. The observations in this study further show that 5% CSE induced higher intracellular levels of TGF-*β*1 and IL-1*β* in GO orbital fibroblasts than those in the normal controls. Moreover, 5% CSE-induced elevation of TGF-*β*1 and IL-1*β* in GO orbital fibroblasts was abolished by the antioxidant treatment. TGF-*β*1, a potent fibrogenic factor, has been reported to modulate the proliferation of fibroblasts and tissue fibrosis [35, 36]. IL-1*β*, an important proinflammatory cytokine in GO, has been shown to stimulate hyaluronan synthesis in orbital fibroblasts [37]. Fibroblast proliferation, tissue fibrosis, and hyaluronan accumulation are all important pathological features in the clinical expression of GO. Collectively, these findings suggest that oxidative stress plays an important role on the deteriorative effect of cigarette smoking on GO.

In conclusion, this study demonstrated that GO fibroblasts have exaggerated response to cigarette smoke extract challenge along with increased oxidative stress, fibrosis-related genes expression, and intracellular levels of TGF-*β*1 and IL-1*β*. The findings obtained in this study provide some clues for the impact of cigarette smoking on GO and offer a theoretical basis for the potential and rational use of antioxidants in treating GO.

## Figures and Tables

**Table 1:** The induction ratio of fibrotic-related genes expression in the orbital fibroblasts from normal subjects and GO patients before and after 5% CSE treatment.

Fibrosis-related genes	Basal levels (mean ± SD)%^a^	5% CSE-treated (mean ± SD)%^a^	Induction ratio (%) (mean ± SD)%	*p* value
*Apolipoprotein J*				
Normal (*n* = 5)	108.36 ± 5.63	135.49 ± 9.74	125.83 ± 11.74	0.0431
GO (*n* = 5)	173.54 ± 6.67	301.67 ± 12.73	168.96 ± 15.47	0.0085
	*p* = 0.0381		*p* = 0.0086	

*Fibronectin*				
Normal (*n* = 5)	105.38 ± 5.83	147.37 ± 9.47	139.84 ± 14.37	0.0318
GO (*n* = 5)	263.45 ± 10.37	484.21 ± 15.87	180.47 ± 16.71	0.0033
	*p* = 0.0046		*p* = 0.0031	

*CTGF*				
Normal (*n* = 5)	117.38 ± 4.22	158.57 ± 8.42	135.09 ± 12.88	0.0441
GO (*n* = 5)	223.07 ± 14.17	379.22 ± 14.85	169.74 ± 11.53	0.0064
	*p* = 0.0074		*p* = 0.0054	

^a^The expression levels from 5 normal subjects and 5 GO patients were normalized to each individual 18S rRNA gene expression followed by adjusting to N1 whose expression was defined as 100%.

**Table 2:** The inhibition ratio of 5% CSE-induced fibrotic-related genes expression by antioxidants in the orbital fibroblasts from GO patients.

Fibrosis-related genes	5% CSE-treated (mean ± SD)%^a^	1 mM NAC + 5% CSE-treated (mean ± SD)%^a^	Inhibition ratio (%) (mean ± SD)	2 mM VitC + 5% CSE-treated (mean ± SD)%^a^	Inhibition ratio (%) (mean ± SD)
*Apolipoprotein J*					
GO (*n* = 5)	285.17 ± 22.47	245.18 ± 20.33	14.02 ± 3.27	217.32 ± 18.53	23.79 ± 8.47
			*p* = 0.0437		*p* = 0.0294

*Fibronectin*					
GO (*n* = 5)	496.35 ± 28.64	412.11 ± 26.72	16.96 ± 4.48	355.38 ± 23.29	28.40 ± 5.05
			*p* = 0.0251		*p* = 0.0085

*CTGF*					
GO (*n* = 5)	350.32 ± 25.73	305.32 ± 23.37	12.82 ± 5.67	254.29 ± 21.77	27.41 ± 9.67
			*p* = 0.0470		*p* = 0.0224

^a^The expression levels from 5 normal subjects and 5 GO patients were normalized to each individual 18S rRNA gene expression followed by adjusting to N1 whose expression was defined as 100%.

**Table 3:** The induction ratio of intracellular cytokine in the orbital fibroblasts from normal subjects and GO patients before and after 5% CSE treatment.

Cytokines species	Basal levels (mean ± SD)	5% CSE-treated (mean ± SD)	Induction ratio (%) (mean ± SD)	*p* value
*TGF-β1 (pg per 10* ^*4*^ * cells)*				
Normal (*n* = 5)	87.46 ± 13.88	117.05 ± 18.11	133.83 ± 10.61	0.041
GO (*n* = 5)	148.67 ± 18.77	282.37 ± 16.29	189.67 ± 12.55	0.006
	*p* = 0.004		*p* = 0.008	

*IL-1β (pg per 10* ^*4*^ * cells)*				
Normal (*n* = 5)	57.05 ± 9.73	68.19 ± 8.33	119.07 ± 7.22	0.033
GO (*n* = 5)	75.83 ± 7.52	122.38 ± 11.37	161.58 ± 9.73	0.005
	*p* = 0.008		*p* = 0.003	

**Table 4:** The inhibition ratio of 5% CSE-induced intracellular cytokine by antioxidants in the GO orbital fibroblasts.

Cytokines species	5% CSE-treated (mean ± SD)	1 mM NAC + 5% CSE-treated (mean ± SD)	Inhibition ratio (%) (mean ± SD)	2 mM VitC + 5% CSE-treated (mean ± SD)	Inhibition ratio (%) (mean ± SD)
*TGF-β1 (pg per 10* ^*4*^ * cells)*					
GO (*n* = 5)	265.91 ± 13.67	177.08 ± 14.05	33.41 ± 12.39	153.39 ± 9.22	42.32 ± 11.25
			*p* = 0.037		*p* = 0.008

*IL-1β (pg per 10* ^*4*^ * cells)*					
GO (*n* = 5)	139.77 ± 8.20	92.83 ± 9.37	33.09 ± 7.33	81.27 ± 7.26	41.85 ± 5.28
			*p* = 0.028		*p* = 0.003
